# Immunological adjuvant effect of the peptide fraction from the larvae of *Musca domestica*

**DOI:** 10.1186/s12906-015-0951-6

**Published:** 2015-12-02

**Authors:** Liqing Chen, Juan Zhang, Hongxiang Sun

**Affiliations:** College of Animal Sciences, Zhejiang University, Hangzhou, 310058 China

**Keywords:** *Musca domestica* larvae, Peptide, Adjuvant, Avian influenza vaccine, Cellular and humoral, Th1/Th2 immune responses

## Abstract

**Background:**

The larvae of *Musca domestica* (Diptera: Muscidae) have been used traditionally for malnutritional stagnation, decubital necrosis, osteomyelitis, ecthyma and lip scald and also to treat coma and gastric cancer in the traditional Chinese medicine. Its immunomodulatory effects in naïve mice in relation to the traditional uses were also reported. However, the immunological adjuvant potentials of this insect have not yet been studied.

**Methods:**

The peptide fraction from the larvae of *Musca domestica* L. (MDPF) was evaluated for its adjuvant potentials on the immune responses to ovalbumin (OVA) and avian influenza vaccine (rL–H5) by determining antigen-specific antibody titers, splenocyte proliferation, activity of natural killer (NK) cell, the secretion of cytokines from splenocytes in the immunized mice.

**Results:**

MDPF significantly enhanced not only the concanavalin A (Con A)-, lipopolysaccharide (LPS)- and antigen-stimulated splenocyte proliferation, but serum antigen-specific IgG, IgG1, IgG2a, and IgG2b antibody titers in the mice immunized with OVA and rL–H5. MDPF also remarkably promoted the killing activities of NK cells in splenocytes from the mice immunized with rL–H5. Furthermore, MDPF significantly promoted the production of Th1 (IL-2 and IFN-γ) and Th2 (IL-10) cytokines from splenocytes in the immunized mice.

**Conclusions:**

The results indicated that MDPF had a potential to increase both cellular and humoral immune responses and elicit a balanced Th1/Th2 response, and that MDPF may be a safe and efficacious vaccine adjuvant candidate.

**Electronic supplementary material:**

The online version of this article (doi:10.1186/s12906-015-0951-6) contains supplementary material, which is available to authorized users.

## Background

Insects and insect derivatives have been widely used in folk medicine across the world since ancient times [1, 2]. At present, there are approximately 300 medicinal insects distributed in 70 genera, 63 families, and 14 orders. An estimated 1700 traditional Chinese medicine prescriptions include medicinal insects or insect-derived crude drugs [3].

*Musca domestica* (housefly) belongs to the order of Diptera. The larvae of *M. domestica* have been clinically used to treat malnutritional stagnation, decubital necrosis, osteomyelitis, ecthyma, and lip scald in traditional Chinese medicine [4]. The main constituents of *M. domestica* larvae include protein, antimicrobial peptides, polyunsaturated fats, polysaccharides, lysozyme, agglutinin, vitamins, and minerals [5]. Among them, antimicrobial peptides such as cecropin, defensin attacin, and MDpep9 have been paid an extensive attention [6–8]. Antimicrobial peptides of *M. domestica* larvae have been shown to possess the antioxidant [9], antitumor [10, 11], anti-inflammatory [12], anti-atherosclerosis [13], hepatoprotective [14], antiviral and immunomodulatory [15] activities.

It was reported that the protein-enriched fraction of *Musca domestica* larvae could promote the phagocytic function of macrophages, 2,4-dinitrofluorobenzene-induced delayed type hypersensitivity reaction, proliferation of lymphocytes, and natural killer cell activity in naïve mice [15]. In our previou works, the peptide fraction from *Musca domestica* larvae (MDPF) was found to improve both specific and non-specific cellular and humoral immune response in tumor-bearing mice, and its antitumor activity might be achieved by switching-on of Th1-based protective cell-mediated immunity [16]. It was recently reported that some antimicrobial host defence peptides from insects had shown excellent vaccine adjuvant properties in mouse models [17]. Although many adjuvants have been proposed over the last few decades, the vast majority have not been successful in being approved for human use, with limitations including unacceptable local or systemic toxicity, manufacturing difficulties, instability, and prohibitive cost [18, 19]. To further the search for a novel, safer, and efficacious adjuvant, therefore, the current study was undertaken to evaluate the adjuvant potential of MDPF on the cellular and humoral immune responses to ovalbumin (OVA) and Newcastle disease virus-based recombinant avian influenza vaccine (rL–H5) in mice.

## Methods

### Materials

Newcastle disease virus-based recombinant influenza vaccine (rL–H5) and H5 subtype avian influenza virus antigen (H5–Ag) were purchased from Harbin Weike Biotechnology Development Company, Heilongjiang, China. OVA, concanavalin A (Con A), lipopolysaccharide (LPS), 3-(4,5-dimethylthiazol-2-yl)-2,5-diphenyltetrazolium bromide (MTT), RPMI-1640 medium, and rabbit anti-mouse IgG peroxidase conjugate were purchased from Sigma Chemical Co., Saint Louis, MO, USA; goat anti-mouse IgG1, IgG2a, and IgG2b peroxidase conjugate were from Southern Biotech. Assoc., Birmingham, AL, USA; cytokine (IL-2, IL-10, and IFN-γ) detecting ELISA kits were from Wuhan Boster Biological Technology Co. Ltd., Hubei, China. Quil A was kindly provided by Brenntag Nordic A/S, Denmark. Fetal calf serum (FCS) was purchased from Hyclone, Utah, USA.

Human leukemia K562 cells, sensitive to natural killer (NK) cells, were purchased from Institute of Cell Biology, Chinese Academy Sciences. They were maintained in the logarithmic phase of growth in RPMI-1640 medium supplemented with 2 mM L-glutamine, 100 IU/ml penicillin, 100 μg/ml streptomycin, and 10 % FCS at 37 °C under humidified air with 5 % CO_2_.

### Preparation and characterization of MDPF

The third instar larvae of *Musca domestica* were collected in Zhejiang Xiangshan Nursery, China in November, 2010. A voucher specimen (No. 20101105) has been deposited at the Laboratory of Nature Drug, College of Animal Sciences, Zhejiang University, China, and identified by professor Jun-An Ye at College of Animal Sciences, Zhejiang University. MDPF were prepared from the third instar larvae of *Musca domestica* and characterized as previously described [16] (Additional file 1). The protein content of MDPF was about 56.24 % ± 3.9 % using bovine serum albumin as the standard. The results of SDS-PAGE showed that the molecular weights of MDPF were ca. 10 kD (Additional file 1: Figure S1). A stock MDPF solution with a concentration of 10 mg/ml was prepared by dissolving in 0.89 % saline. The solution was sterilized by passing it through a 0.22-μm Millipore filter, and then analyzed for endotoxin level by a gel-clot *Limulus* amebocyte lysate assay.

### Experimental animals

Female ICR mice (Grade II, 5 weeks old) weighing 18–22 g were purchased from Zhejiang Experimental Animal Center (Certificate No. SCXK 2008‐0033, Hangzhou, China), and acclimatized for 1 week prior to use. Rodent laboratory chow and tap water were provided ad libitum and maintained under controlled conditions with a temperature of 24 ± 1 °C, humidity of 50 ± 10 %, and a 12/12-h light/dark cycle. All the procedures were in strict accordance with the PR China legislation on the use and care of laboratory animals and with the guidelines established by Institute for Experimental Animals of Zhejiang University and were approved by the University Committee for Animal Experiments.

### Toxicity assays

Six-week-old female ICR mice were divided into groups, each consisting of five mice. Animals were injected twice subcutaneously (*s.c*.) on the back with MDPF at a single dose of 0.5, 1.0, 2.5, and 5.0 mg in 0.5 ml saline solution at weekly intervals, and monitored daily for 14 days. The toxicities of Quil A were also determined with one preparation in this study. Varying doses (100, 150, 200 μg) of Quil A dissolved in saline were injected *s.c*. in mice. Saline-treated animals were included as control group. The toxicity was assessed by lethality, local swelling and loss of hair at the site of injection.

### Immunization

To investigate the adjuvant potentials on the immune responses to OVA, ICR mice were randomly divided into six groups, each consisting of six mice. Animals were subcutaneously (*s.c*.) injected twice with OVA 25 μg alone or with OVA 25 μg dissolved in saline containing Quil A (10 μg) or MDPF (10, 25, or 50 μg) at 2-week intervals. Saline-treated animals were included as controls. Sera and splenocytes were collected 14 days after the boosting immunization for measurement of OVA-specific antibody titers, splenocyte proliferation, and cytokine levels.

To evaluate the adjuvant effects of MDPF on rL–H5 vaccine, mice were randomly divided into six groups, with eight mice per group. Animals were immunized *s.c.* twice with rL–H5 (10^6^ EID_50_/dose) alone or in combination with Quil A (10 μg), or MDPF (100, 200, or 300 μg) in 0.2 ml saline on day 1. A boosting injection was given 2 weeks later. Saline-treated animals were included as controls. Sera and splenocytes were collected 2 weeks after the second immunization for measurement of antigen-specific antibody titers, splenocyte proliferation, NK cell activity and cytokine levels.

### Splenocyte proliferation assay

Splenocytes were prepared 2 weeks after the secondary immunization, and cultured with Con A (final concentration 5 μg/ml), LPS (final concentration 10 μg/ml), OVA (final concentration 20 μg/ml), H5–Ag (final concentration 0.125 hemagglutinating units (HAU)/ml), or RPMI-1640 medium at 37 °C in a humid atmosphere with 5 % CO_2_ for 48 h. Splenocyte proliferation was measured by the MTT method as previously described [20]. The stimulation index (SI) was calculated based on the following formula: SI = the absorbance value for mitogen-cultures divided by the absorbance value for non-stimulated cultures.

### Assay of NK cell activity

Splenocytes were prepared as effector cells 2 weeks after the boosting immunization. The activity of NK cells in splenocytes was measured by the MTT assay using human leukemia K562 cell lines as target cells as previously xdescribed [21]. Three kinds of control measurements were performed: target cells control, blank control and effector cells control. NK cell activity was calculated as following equation: NK activity (%) = (OD_T_ − (OD_S_ − OD_E_))/OD_T_ × 100, where OD_T_, absorbance of target cells control, OD_S_, absorbance of test samples and OD_E_, absorbance of effector cells control.

### Measurement of serum antigen-specific IgG antibody and its isotype titers

OVA- or H5–Ag-specific IgG, IgG1, IgG2a, and IgG2b antibodies in sera were detected in individual serum sample by an indirect ELISA as previously described [22]. The absorbance was measured in an ELISA reader at 492 nm, where sets of sera samples have been subjected to within and between group comparisons. ELISA assays were performed on the same day for all of the samples.

### Cytokine measurements

Splenocytes (5 × 10^6^ cells/well) from the immunized mice prepared as described above were incubated with Con A (final concentration 5 μg/ml) in 24-well culture plates at 37 °C in 5% CO_2_. After 48 h, the plate was centrifuged at 1400 × *g* for 5 min and culture supernatants were collected for the detection of IL-2, IL-10, and IFN-γ levels using commercial ELISA kits [20]. The concentrations of IL-2, IL-10, and IFN-γ were calculated according to the standard curve using each of the recombinant cytokines in the ELISA kits.

### Statistical analysis

The data were expressed as mean ± standard deviation (SD) and examined for their statistical significance of difference with ANOVA and a Tukey post hoc test. *P*-values of less than 0.05 were considered to be statistically significant.

## Results

### Toxicity of MDPF

The endotoxin level in a stock MDPF solution with a concentration of 10 mg/ml was measured to be less than 0.5 endotoxin units (EU)/ml. Therefore, the MDPF sample used in this study was excluded from endotoxin contamination. When the animals were administered *s.c*. twice at the dose up to 5.0 mg at weekly intervals, there is no lethality observed. Local swelling or loss of hair was not observed in mice at the tested doses. In addition, no MDPF-spcific antibody was detected in the sera from all the immunized mice in this study. The results suggested that the safety dose of MDPF used for mice was at least up to 200 mg/kg. However, the local swelling and loss of hair were found at the site of injection in the mice received Quil A at three doses. Furthermore, the numbers of deaths per group of five mice were 2 and 3 within 72 h after subcutaneous injection of Quil A at the doses of 150 and 200 μg with one preparation.

### Effect of MDPF on splenocyte proliferation

**Fig. 1:**
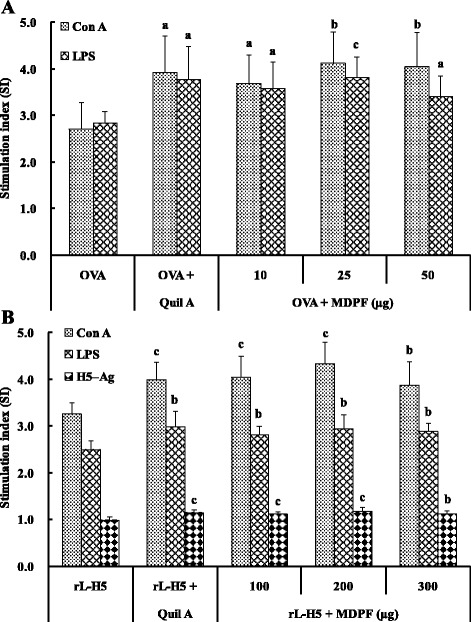
Effect of MDPF on mitogen- and antigen-stimulated splenocyte proliferation in the mice immunized with OVA **a** and rL–H5 **b**. Splenocyte proliferation was measured by the MTT method and shown as a stimulation index (SI). The values are presented as means ± SD (*n* = 6 or 8). Significant differences with OVA alone or rL–H5 alone group were designated as ^*a*^ *P* < 0.05, ^b^ *P* < 0.01, and ^c^ *P* < 0.001

The effects of MDPF on mitogen-stimulated splenocyte proliferation in the mice immunized OVA were shown in Fig. 1a. Con A- and LPS-stimulated splenocyte proliferation in the mice immunized with OVA/Quil A and OVA/MDPF (10, 25, and 50 μg) was significantly higher than that in the OVA alone group (*P* < 0.05, *P* < 0.01 or *P* < 0.001). As shown in Fig. 1b, Quil A and MDPF (100, 200, and 300 μg) also significantly promoted Con A- and LPS-stimulated splenocyte proliferation in the mice immunized with rL–H5 (*P* < 0.01 or *P* < 0.001). H5–Ag-induced splenocyte proliferation in mice immunized with rL-H5/Quil A and rL–H5/MDPF (100, 200, and 300 μg) was also greater than that observed for the mice immunized with rL–H5 alone (*P* < 0.01 or *P* < 0.001).

### Effects of MDPF on NK cell activity

**Fig. 2:**
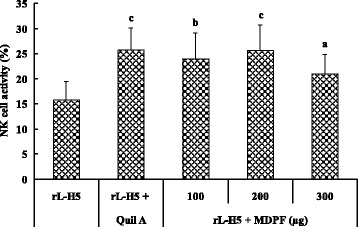
Effect of MDPF on NK cell activity in the splenocytes from the mice immunized with rL–H5. Splenocytes were prepared 2 weeks after the secondary immunization, and assayed for NK cell activity by the MTT assay. The values are presented as means ± SD (*n* = 8). Significant differences with rL–H5 alone group were designated as ^*a*^ *P* < 0.05, ^b^ *P* < 0.01, or ^c^ *P <* 0.001

The effects of MDPF on NK cell activity in mice immunized with rL–H5 were shown in Fig. 2. Quil A and MDPF significantly increased the killing percentage of NK cells in splenocytes from the rL–H5-immunized mice against K562 cells compared with rL-H5 vaccine alone group (*P* < 0.05, *P* < 0.01, or *P* < 0.001), indicating that MDPF could promote NK cell lytic activities in the mice immunized with rL–H5.

### Effect of MDPF on the serum antigen-specific antibody response

**Fig. 3:**
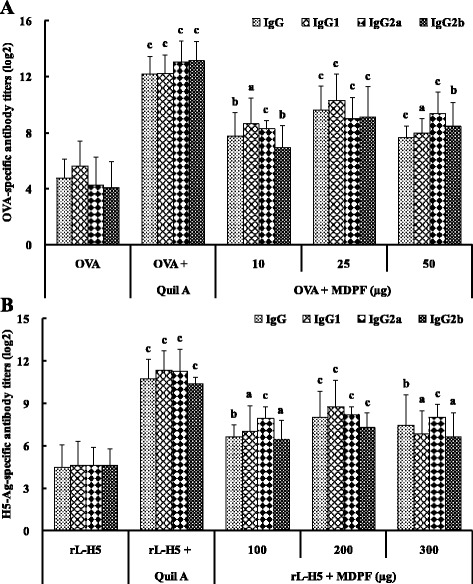
Effect of MDPF on antigen-specific IgG and its isotype titers in the mice immunized with OVA **a** and rL–H5 **b**. Antigen-specific IgG, IgG1, IgG2a, and IgG2b antibodies in the sera were measured by an indirect ELISA. The values are presented as means ± SD (*n* = 6 or 8). Significant differences with OVA alone or rL–H5 alone group were designated as ^*a*^ *P* < 0.05, ^b^ *P* < 0.01, and ^c^ *P* < 0.001

The effect of MDPF on the serum OVA-specific IgG and its isotype titers in the immunized mice were shown in Fig. 3a. Quil A and MDPF (10, 25, and 50 μg) significantly increased the OVA-specific serum IgG and IgG1 titers in OVA-immunized mice (*P* < 0.05, *P* < 0.01, or *P* < 0.001). Significant enhancements in OVA-specific serum IgG2a and IgG2b antibody titers were also observed in the mice immunized with OVA/Quil A and OVA/MDPF compared with OVA alone group (*P* < 0.01 or *P* < 0.001). Similarly, the serum H5–Ag-specific IgG, IgG1, IgG2a, and IgG2b antibody titers in rL–H5-immunized mice were also markably enhanced by Quil A and MDPF (*P* < 0.05, *P* < 0.01 or *P* < 0.001) (Fig. 3b). The findings indicated that MDPF significantly promted the humoral immune responses in OVA- and rL–H5-immunized mice.

### Effect of MDPF on cytokine secretion by splenocytes

**Fig. 4:**
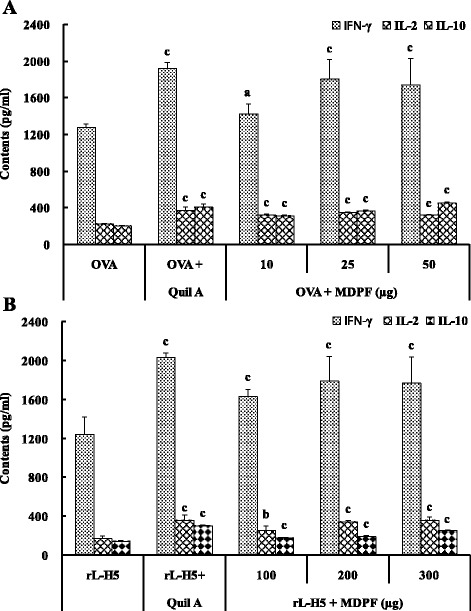
Effects of MDPF on cytokine production from splenocytes in the mice immunized with OVA **a** and rL–H5 **b**. Splenocytes were incubated with Con A for 48 h, and the culture supernatants were collected for the detection of IL-2, IL-10, and IFN-γ levels using commercial ELISA kits. The values are presented as means ± SD (*n* = 6 or 8). Significant differences with OVA alone or rL–H5 alone group were designated as ^*a*^ *P* < 0.05, ^b^ *P* < 0.01, and ^c^ *P* < 0.001

The calibration curves of IL-2, IFN-γ, and IL-10 were constructed with mouse cytokine standards, and their correlation coefficients were all bigger than 0.9980. As shown in Fig. 4a, the contents of cytokines IL-2, IFN-γ, and IL-10 in the supernatants from cultured splenocytes in the mice immunized with OVA/Quil A and OVA/MDPF were significantly higher than those in the mice immunized with in OVA alone (*P* < 0.05 or *P* < 0.001). Quil A and MDPF also significantly promoted the secretion of cytokines IL-2, IFN-γ, and IL-10 from the splenocytes in the mice immunized with rL–H5 (*P* < 0.01 or *P* < 0.001) (Fig. 4b).

## Discussion

Vaccines are one of the most successful medical interventions for the prevention of infectious diseases. New vaccine technology has led to vaccines containing highly purified antigens with improved tolerability and safety profiles, but the immune response they induce is suboptimal without the help of adjuvants [23]. The use of novel adjuvants in combination with the immunogen holds great promise towards the goal of enhancing the potency, breadth and durability of vaccines [24]. The strong adjuvant activity is often correlated with increased toxicity. Among nature products, saponins-based adjuvants, especially Quil A from the bark of *Quillaja saponaria*, have been tested widely in novel vaccine design [25]. The unique capacity of Quil A to stimulate both the Th1 immune response and the production of cytotoxic T-lymphocytes against exogenous antigens makes it ideal for use in subunit vaccines and vaccines directed against intracellular pathogens as well as for therapeutic cancer vaccines [26]. However, in addition to pain on injection, severe local reactions and granulomas, toxicity includes severe haemolysis [27] making Quil A unsuitable for human uses other than for life threatening diseases, such as HIV infection or cancer [28]. In addition, the saponins have a strong adjuvant activity when administered parenterally, in general, while they have a low or no activity when delivered orally [29]. The development of a safe, novel and potent adjuvant with the ability to enhance and direct broad and durable immune responses to these otherwise poorly immunogenic antigens is hence a top priority [30, 31].

It was reported that the oral maximum tolerated dose of *Musca domestica* larvae extracts (MDLE), consisted of protein fraction and chitosan at the ratio of 10–1, was more than 33.0 g/kg body weight for mice [32, 33]. The systemic evaluation of the subchronic toxicity in Sprague Dawley rats indicated that no observed adverse effect level for MDLE is 13.2 and 33.0 g/kg bw/day following a 13-week repeated dose in male and female rats, respectively [34]. MDPF used in this study was not found to cause any side effects, toxicity, and mortality in the mice injected *s.c*. twice at the dose up to 5.0 mg at weekly intervals. In this study, however, Quil A was found to exhibited severe side effects, toxicity, and lethalities. The lethalities of mice were 40 % and 60 % within 72 h after subcutaneous injection of Quil A at the doses of 150 and 200 μg with one preparation, respectively. Kensil et al. [35] reported that the Quil A was lethal to CD-1 mice in the dose range of 100–125 μg. The results are consistent with our findings about Quil A.

Meanwhile, no MDPF-spcific antibody was also detected in all the mice in this study. These results suggested that the MDPF is much safer in clinical use for human and animals. In addition, antimicrobial peptides could exert the similar pharmacological effects when administered parenterally and orally. In our previous work, the oral administration of MDPF could significantly not only enhance splenocytes proliferation, NK cell and CTL activity, but also promote the production of IFN-γ and up-regulate the mRNA expression levels of IFN-γ and Th1 transcription factors T-bet and STAT-4 in splenocytes from the S180-bearing mice [16]. To further search a safe, novel and potent adjuvant, in this study, MDPF was evaluated for the adjuvant potential on the immune responses to OVA and rL–H5 in mice.

The cellular immune response plays an important role in the generation of both humoral and cell-mediated responses to vaccination. The mobilized T lymphocytes proliferate to protect the body by activating other immune cells or by killing infected cells. Among the immune cells activated by T cells, most importantly, are the B lymphocytes that produce antibodies. T lymphocytes direct the types of antibodies that B cells produce and the activity of other immune cells, thereby directing the immune response to optimally provide protection against different types of infections [36]. The capacity to elicit an effective T- and B-lymphocyte immunity can be shown by the stimulation of lymphocyte proliferation response [37]. It is generally known that Con A stimulates T cell proliferation and LPS stimulates B cell proliferation. The proliferation assay showed that MDPF could significantly promote the Con A-, LPS-, and H5–Ag-stimulated splenocyte proliferation in the immunized mice (Fig. 1). These results indicated that MDPF was effective in inducing strong activation potential of T and B cells in OVA- and rL–H5-immunized mice, and that MDPF showed adjuvant potentials on the cell-mediated immunity induced by non-specific or antigen-specific stimulation.

NK cells represent a major population of cytotoxic lymphocytes, and are important in the defense against tumors and viruses [38]. With spontaneous cell-mediated cytotoxicities, NK cells are functionally similar to CTLs. NK cells are able to deliver a response immediately after recognizing specific signals, including stress signals, ‘danger’ signals or signals from molecules of foreign origin [39]. In this investigation, MDPF significantly enhanced the lytic activity of NK cells in mice immunized with rL–H5 (Fig. 2), suggesting that the usage of MDPF in rL–H5 could help to improve cytolytic activities against avian influenza virus.

Humoral immunity functions by neutralizing and eliminating extracellular microbes and microbial toxins. In addition to neutralizing properties, antibodies can mediate host effector functions and facilitate the removal of a pathogen from a host. Therefore, the adjuvant activity of MDPF on serum antigen specific IgG and its isotype antibody responses to OVA and rL–H5 were also evaluated. In this study, MDPF significantly not only enhanced OVA- and H5–Ag-specific IgG and IgG1 titers, but increased specific IgG2a and IgG2b antibody titers in immunized mice (Fig. 3). These results clearly demonstrated that MDPF modulated the quality of immune responses, and elicited a balanced Th1/Th2 immune response to OVA and rL–H5 in mice as associated sensitively with an enhancement of IgG2a, IgG2b and IgG1 levels [40].

Evidence now exists to clearly suggest that Th1 or Th2 responses, generated upon antigenic stimulation, can be modulated in vivo depending on the adjuvant used for immunization [41]. The different Th1 and Th2 immune response profiles correspond to the activation of two distinct major subsets of T-cells characterized by their pattern of cytokine production [42]. The Th1 immune response is characterized by the production of cytokines IL-2, TNF-β and IFN-γ, and an enhanced production of IgG2a, IgG2b and IgG3 in mice. The Th2 response is characterized by the production of cytokines IL-4, IL-5 and IL-10, and an enhanced production of IgG1 and secretary IgA [43]. Immunity to different infectious agents requires distinct types of immune responses. The Th1 response, correlated with the induction of cell-mediated immunity [44], is required for protective immunity against intracellular infectious agents, such as viruses, certain bacteria and protozoa, and presumably against cancer cells [45]. Th2 immunity, which controls the humoral immune response through the triggering of B cell proliferation and differentiation [46], is effective for protection against most bacterial as well as certain viral infections [47]. In order to clearly establish that Th cell-derived cytokines were involved in the adjuvant activity of MDPF, we further analyzed the Th1/Th2 cytokine secretion profiles from splenocyte in the mice immunized OVA and rL–H5 vaccine using ELISA. MDPF not only significantly increased the production of Th2 cytokines IL-10, but also strongly enhanced the production of Th1 cytokines IL-2 and IFN-γ from splenocytes in the immunized mice (Fig. 4). These results further confirmed that MDPF would simultaneously improve Th1 and Th2-type responses to OVA and rL–H5 in mice.

Host defence peptides (HDPs) exhibit a wide range of functions ranging from direct antimicrobial properties to immunomodulatory effects. HDPs are critical effectors of both innate and adaptive immunity [48]. A range of antimicrobial HDPs, including defensins and LL-37, might be effective adjuvants as a result of their ability to modulate DC function and antigen-specific immune responses [49], and to elicit the recruitment, differentiation and activation of effector cells at the site of infection [50]. Whether MDPF exert the adjuvant action by affecting the local immune microenvironment at the site of injection is being explored.

The structures of HDPs can be broadly classified as amphipathic α-helix, β-sheet structures with disulphide bonds, extended structures, and loop structures with one disulphide bond [48]. The antimicrobial peptides in *M. domestica* were previously reported to be mostly strong alkaline [8, 51–54]. However, some weak acidic antibacterial peptides were also isolated from *M. domestica* larvae [55, 56], suggesting the complicate nature of antibacterial peptides in *Musca domestica* larvae. Our studies will be continuing isolation, purification and characterization of the antibacterial peptides in MDPF, and clarification of the relationship between thir adjuvant activity and structure.

In conclusion, the present study demonstrated that MDPF had immunological adjuvant activity on specific cellular and humoral immune responses to OVA and rL–H5 in mice and could simultaneously elicit a Th1 and Th2 immune response. In light of its low or non-existent toxicity to humans and animals, and long-standing use as folk medicines, MDPF may be a safe and efficacious adjuvant candidate suitable for vaccines for which a balanced and potent stimulation of both the cellular and humoral responses is required. The finding could provide a reference for the further development of *Musca domestica* larvae.

## Additional file

Additional file 1:
**Extraction, purification and characterization of MDPF.** (DOC 243 kb)
