# Supplementary material for: Immunological adjuvant effect of the peptide fraction from the larvae of Musca domestica
Source: BMC Complement Altern Med. 2015 Dec 2;15:427. doi: 10.1186/s12906-015-0951-6 (PMC4668601; doi:10.1186/s12906-015-0951-6)
Supplement: Additional file 1: — Extraction, purification and characterization of MDPF. (DOC 243 kb) [file 12906_2015_951_MOESM1_ESM.doc]

Title: Immunological adjuvant effect of the peptide fraction from the larvae of *Musca domestica*

Authors: Chen LQ, et al.

**Supplementary material**

**Extraction, purification and characterization of MDPF**

The third instar larvae of *Musca domestica* were collected in Zhejiang Xiangshan Nursery, China in November, 2010. A voucher specimen (No. 20101105) has been deposited at the Laboratory of Nature Drug, College of Animal Sciences, Zhejiang University, China. MDPF were isolated and purified from the larvae of *Musca domestica* as previously described (Guo ZY, Shi YH, Le GW. Adsorption and separa tion of *Musca domestica* antimicrobial peptide with macroporous adsorption resin. Natural Product Research and Development 2008;20: 969–973). Briefly, the collected fresh larva were washed with distilled water, dried with absorbent paper and then frozen to death at –20 °C. 300 g larva were weighed and homogenized with 600 ml of 3% acetic acid (HAc) for 5 min in a glass beaker using a high speed tissue gravity mill. The homogenate was stored overnight at 4 °C, centrifuged at 4000 rpm for 10 min at 4 °C. The resulting precipitate was treated once again with the same method. The combined supernatant was heated at 100 °C for 1 h, stored overnight at 4 °C for 3 h, and then filtered through filter paper. The filtrate was lyophilized to afford a total extract (26.27 g). The dried extract (17.50 g) was subjected to D101 resin column chromatography, washed with H_2_O, and eluted with 55% ethanol (EtOH). The collected eluates were concentrated and lyophilized to afford a peptide-enriched fraction (MDFP). Protein concentration in MDPF was determined using bicinchoninic acid (BCA) protein assay kit according to the manufacturer׳s instructions. Sodium dodecyl sulfate polyacrylamide gel electrophoresis (SDS-PAGE) of MDFP was performed on 5.0% stacking gel and a 12.5% separation gel.

Title: Immunological adjuvant effect of the peptide fraction from the larvae of *Musca domestica*

Authors: Chen LQ, et al.

**Supplementary Fig. 1.**

| **1** | **2** | **3** | **M** |  |
| --- | --- | --- | --- | --- |
| 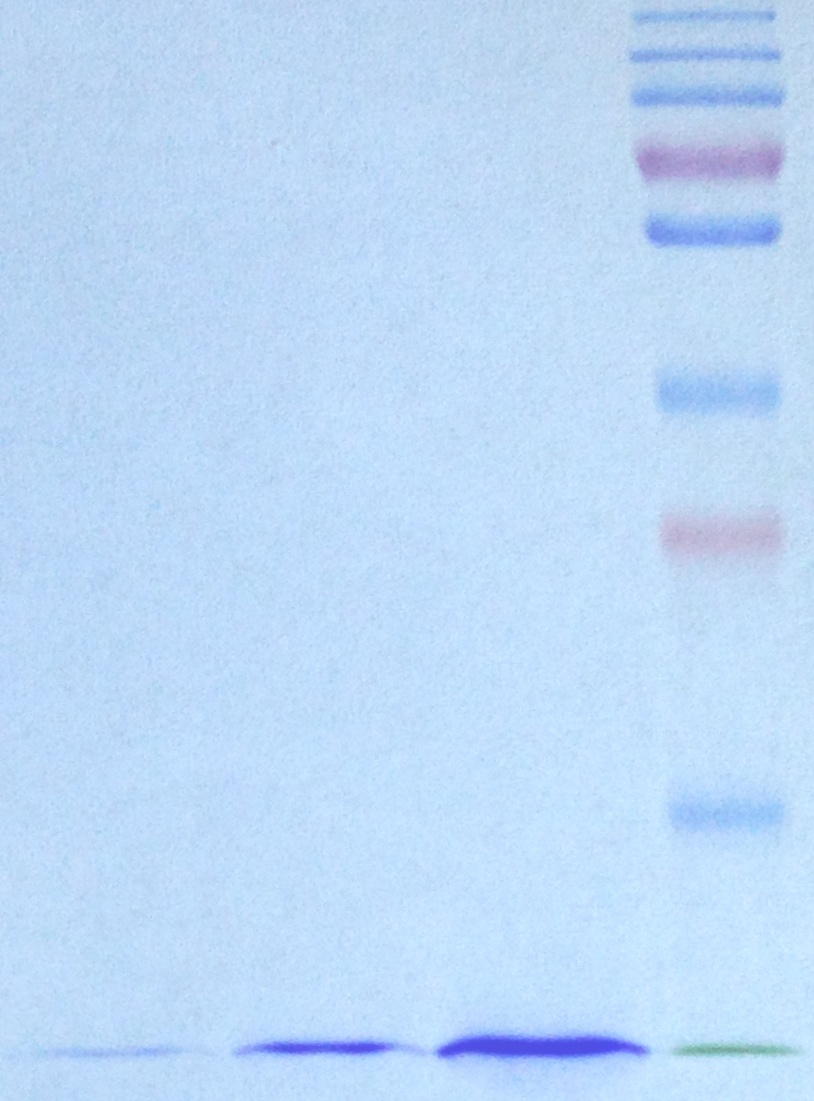 | | | | **250 kD** |
|  |  |  |  | **130 kD** |
|  |  |  |  | **100 kD** |
|  |  |  |  | **70 kD** |
|  |  |  |  | **55 kD** |
|  |  |  |  | **35 kD** |
|  |  |  |  | **25 kD** |
|  |  |  |  | **15 kD** |
|  |  |  |  | **10 kD** |

SDS-PAGE analysis of the peptide fraction from *Musca domestica* larvae (MDPF). Lane 1, MDPD (0.5 mg/ml); Lane 2, MDPD (1 mg/ml); Lane 3, MDPD (2 mg/ml); M, molecular mass marker.
